# Feed additives decrease survival of delta coronavirus in nursery pig diets

**DOI:** 10.1186/s40813-016-0048-8

**Published:** 2017-01-20

**Authors:** Katie M. Cottingim, Harsha Verma, Pedro E. Urriola, Fernando Sampedro, Gerald C. Shurson, Sagar M. Goyal

**Affiliations:** 1 Department of Animal Science, University of Minnesota, St. Paul, MN 55108 USA; 2 Department of Veterinary Population Medicine, University of Minnesota, St. Paul, MN 55108 USA

**Keywords:** Feed additives, Inactivation kinetics, Porcine delta coronavirus, Survival, Swine, Transmission, Virus

## Abstract

**Background:**

Feed contaminated with feces from infected pigs is believed to be a potential route of transmission of porcine delta coronavirus (PDCoV). The objective of this study was to determine if the addition of commercial feed additives (e.i., acids, salt and sugar) to swine feed can be an effective strategy to inactive PDCoV.

**Results:**

Six commercial feed acids (UltraAcid P, Activate DA, KEMGEST, Acid Booster, Luprosil, and Amasil), salt, and sugar were evaluated. The acids were added at the recommended concentrations to 5 g aliquots of complete feed, which were also inoculated with 1 mL of PDCoV and incubated for 0, 7, 14, 21, 28, and 35 days. In another experiment, double the recommended concentrations of these additives were also added to the feed samples and incubated for 0, 1, 3, 7, and 10 days. All samples were stored at room temperature (~25 °C) followed by removal of aliquots at 0, 7, 14, 21, 28, and 35 days. Any surviving virus was eluted in a buffer solution and then titrated in swine testicular cells. Feed samples without any additive were used as controls. Both Weibull and log-linear kinetic models were used to analyze virus survival curves. The presence of a tail in the virus inactivation curves indicated deviations from the linear behavior and hence, the Weibull model was chosen for characterizing the inactivation responses due to the better fit. At recommended concentrations, delta values (days to decrease virus concentration by 1 log) ranged from 0.62–1.72 days, but there were no differences on virus survival among feed samples with or without additives at the manufacturers recommended concentrations. Doubling the concentration of the additives reduced the delta value to ≤ 0.28 days (*P* < 0.05) for all the additives except for Amasil (delta values of 0.86 vs. 4.95 days). Feed additives that contained phosphoric acid, citric acid, or fumaric acid were the most effective in reducing virus survival, although none of the additives completely inactivated the virus by 10- days post-inoculation.

**Conclusions:**

Commercial feed additives (acidifiers and salt) may be utilized as a strategy to decrease risk of PDCoV in feed, specially, commercial feed acidifiers at double the recommended concentrations reduced PDCoV survival in complete feed during storage at room temperature. However, none of these additives completely inactivated the virus.

## Background

There are three enteric coronaviruses that can cause gastrointestinal illness in young pigs e.g., transmissible gastroenteritis virus (TGEV), porcine epidemic diarrhea virus (PEDV), and porcine delta coronavirus (PDCoV) [1]. Transmissible gastroenteritis virus has been present in the United States since 1946, but PEDV and PDCoV were introduced more recently in 2013 and 2014, respectively. The spread of PEDV among swine herds was rapid; and strict biosecurity measures known to prevent transmission of other viruses such as porcine respiratory and reproductive syndrome virus were ineffective; later contaminated complete feed was demonstrated to be a route for PEDV transmission that has been overlooked in previous biosecurity protocols [2]. Therefore, for disease prevention purposes, it is essential to understand proper feed handling procedures that minimize risk of transmission, and to identify methods that can rapidly inactivate these viruses if present in feed.

Commercial swine feed is often fortified with various additives, including acidifiers such as organic and/or inorganic acids to control bacterial and mold growth in feed, increase growth performance of animals, improve nutrient digestibility, and control harmful bacteria in the animal gut [3]. Acidifiers are often added to feed as an alternative to the use of antibiotics as growth promoters and to control pathogens such as *Salmonella* spp. [4, 5]. Nursery pigs are believed to obtain the greatest benefit from the addition of acidifiers, and the addition of acidifiers has been shown to increase growth rate by 12% [6]. Acidifiers are also effective in reducing diarrhea and mortality while maintaining adequate growth of nursery pigs [6]. This study was conducted to determine if the addition of commercially available feed additives (salt, sugar, and acidifiers), at recommended or double the recommended concentrations, is effective in reducing the survival of PDCoV in feed.

## Methods

### Virus propagation

The strain of PDCoV was obtained from the National Veterinary Services Laboratory (NVSL; Ames, IA). Stock virus was propagated in swine testicular cells. The cells were grown in Minimum Essential Medium with Earle’s salts supplemented with L-glutamine (Mediatech, Herndon, VA), 8% fetal bovine serum (Hyclone, South Logan, UT), 50 μg/mL gentamicin (Mediatech), 150 μg/mL neomycin sulfate (Sigma, St. Louis, MO), 1.5 μg/mL fungizone (Sigma), and 455 μg/mL streptomycin (Sigma). The maintenance medium included 5 μg/mL of trypsin (Gibco, Life technologies, Grand Island, NY) and the same antibiotics as previously described. Cells inoculated with the virus were incubated at 37 °C under 5% CO_2_ and were observed for the appearance of virus-induced cytopathic effects (CPE) for up to 6 days post-infection. The infected cells were subjected to 3 freeze-thaw cycles (−80 °C/25 °C) followed by centrifugation at 2500 × g for 15 min at 4 °C. The supernatant was collected, aliquoted, and stored at −80 °C until use.

### Virus titration

Serial 10-fold dilutions of all samples were prepared in maintenance medium followed by inoculation in monolayers of swine testicular cells contained in 96-well microtiter plates (Nunc, NY, USA) using 100 μL/well and 3 wells per dilution. Inoculated cells were incubated at 37 °C under 5% CO_2_ for up to 6 days and examined daily under an inverted microscope for the appearance of CPE. The highest dilution showing CPE was considered the end point. Virus titers were calculated as Tissue Culture Infectious Dose TCID_50_/mL by the Karber method [7].

### Feed matrix and laboratory analysis

The CGI Enhance ground commercial starter feed used in this experiment was obtained from VitaPlus (Madison, WI). This feed is designed for feeding pigs from 5–10 days post-weaning and does not contain any animal derived by-products. The feed was confirmed to be negative for PDCoV by real time reverse transcription-polymerase chain reaction (RT-PCR). A sample of the feed was submitted to Minnesota Valley Testing Laboratories (New Elm, MN), where dry matter (DM; method 930.15), ether extract (method 2003.05), crude protein (CP; method 990.03), crude fiber (method 920.39), and ash (method 942.05) were analyzed following standard procedures [8]. The chemical analysis results of the feed were 91.43% DM, 4.47% EE, 24.2% CP, 2.02% crude fiber, and 9.45% ash on as is basis.

### Feed additives

Six commercial feed acidifiers, UltraAcid P, (Nutriad, Dendermonde, Belgium), Activate DA (Novus International, St. Charles, MO), Acid Booster (Agri-Nutrition, DeForest, WI), Kemgest (Kemin Agrifoods, Des Moines, IA), Luprosil (BASF, Florham Park, NJ), and Amasil (BASF, Florham Park, NJ) were evaluated when added at their manufacturers’ recommended concentrations (Table 1). In addition, the effect of sodium chloride and sucrose on virus survival was also evaluated. In a second experiment, PDCoV survival was evaluated by adding the double of the recommended amounts of these feed additives.

**Table 1:** Commercial name of feed additives, active ingredients, concentration when mixed with complete feed at the manufacturers’ recommended doses (1×) and twice the manufacturers’ recommended doses (2×) along with pH of the diet and additive mixture

Feed additive (Manufacturer); *(Active ingredients)*	Amount		pH^1^	
	1×	2×	1×	2×
Complete feed	0	0	5.82^c^ ± 0.02	5.82^c^ ± 0.02
UltraAcid P (Nutriad, Dendermonde, Belgium); *(orthophosphoric, citric, fumaric, and malic acids)*	150 mg	300 mg	5.84^c^ ± 0.03	5.78^c^ ± 0.02
Acid Booster (Agri-Nutrition, DeForest, WI); *(phosphoric, citric, and lactic acids)*	10 mg	20 mg	5.84^c^ ± 0.02	5.84^cg^ ± 0.05
KEMGEST (Kemin Agrifoods, Des Moines, IA); *(phosphoric, fumaric, lactic, and citric acid)*	10 mg	20 mg	4.20^e^ ± 0.03	3.98^e^ ±0.03
Activate DA (Novus International, St. Charles, MO); *(fumaric, benzoic, and 2-hydroxy-4-methylthiobutanoic acids)*	20 mg	40 mg	5.50^b^ ± 0.03	5.11^b^ ± 0.02
Luprosil (Propionic acid, BASF, Florham Park, NJ); *(99.5% propionic acid)*	56 μl	112 μl	5.74^d^ ± 0.03	5.67^d^ ± 0.03
Amasil (Formic Acid, BASF, Florham Park, NJ); *(61% formic acid, 20.5% sodium formate, 18.5% water)*	46 μl	92 μl	5.88^c^ ± 0.03	5.88^gh^ ± 0.01
Sugar (Shoppers Value, Eden Prairie, MN); *(sucrose)*	20 mg	40 mg	3.22^f^ ± 0.04	2.93^f^ ± 0.02
Salt (Essential Every-day, Eden Prairie, MN); *(sodium chloride)*	20 mg	40 mg	4.93^a^ ± 0.05	4.39^a^ ± 0.03

^1^Results shown are means of three replications; different superscripts differ at (*P* < 0.05)

### Virus inoculation procedure

Forty-eight aliquots of feed (5 g/aliquot) were placed in plastic scintillation vials and the recommended concentrations of each feed additive were added. There were a total of 8 observations at each of the 6-time point for each of the 9 dietary combinations (control and 8 additives). Another set of 40 aliquots of feed were used at double of the recommended concentrations of the additives, for a total of 8 replications per each of the 5-time points and 9 dietary combinations (Table 1). Subsequently, 1 mL of PDCoV (initial titer 3.2 × 10^5^ TCID_50_/mL) was added to all vials. The control treatment consisted of vials containing feed and virus but no feed additive. The samples were thoroughly mixed using a vortex mixer and stored at room temperature (~25 °C). An individual vial served as the experimental unit, and one vial from each set was removed at 0, 7, 14, 21, 28, and 35 days to determine the degree of virus inactivation. In the experiment involving double the recommended concentrations of additives, samples were removed and evaluated for virus inactivation at 0, 1, 3, 7, and 10 days. Different time points were selected to account for greater virus inactivation in the early stages of inoculation. To determine the amount of virus inactivation at each time point, the surviving virus in each vial was eluted by adding 10 mL of 3% beef extract-0.05 M glycine solution at pH 7.2. After thorough mixing by vortexing, the vials were centrifuged at 2500 × g for 15 min. Serial 10-fold dilutions of the supernatants (eluates) were inoculated in swine testicular cells as previously described for virus titration. The amount of surviving virus was calculated and compared with that in control vials (no additive) and was expressed as log_10_ TCID_50_/mL. All treatments were applied and analyzed in triplicate.

### Measurement of pH

Fifty mL of distilled water was added to 5 g of feed contained in a 100 mL glass flask. The feed suspension was stirred at room temperature for 2 h using a magnetic stirrer. The pH was measured using a pH probe (Fisher Scientific, Waltham, MA) at 0, 15, 30, 60, and 120 min. The final pH value was calculated as the average of the values at different time intervals. The average pH for feed was 5.82 ± 0.02 and this value was used to compare the pH values after the addition of feed additives.

### Mathematical models

Inactivation kinetics data (log TCID_50_/mL) were analyzed by using GInaFIT software, a freeware add-on for Microsoft Excel (Microsoft, Redmond, WA) [9]. The traditional log-linear model developed by Bigelow and Esty (1920) was used to characterize the survival curves of PDCoV by using the following equation [10]:

1 $$ \mathrm{Log}\ \mathrm{N} = \mathrm{Log}\ {N}_0 - \left(\mathrm{k} \times \mathrm{t}\right) $$

where *N* is the amount of surviving virus after treatment, *N*_*0*_ is the initial virus titer, *k* is the kinetic parameter (day^−1^), and *t* is the treatment time (d). The kinetic parameter *k* is usually expressed as *D*, which is also known as ‘decimal reduction time’ (time required to reduce initial virus titer by 90% or 1 log at a certain temperature) and was calculated as:

2 $$ \mathrm{D} = \kern0.75em \frac{2.3}{k} $$

The Weibull distribution function has been used to describe non-linear inactivation patterns of different microorganisms after thermal and non-thermal processing. Assuming that the temperature resistance of the virus is governed by a Weibull distribution, Mafart et al. [11] developed the following equation [12]:

3 $$ Log(N)= \log \left({N}_0\right)-{\left(\frac{t}{\delta}\right)}^n $$

where *N* is the surviving virus titer after treatment, *N*_*0*_ is the initial virus titer, *δ* is the time (min or days) of first logarithm decline in virus titer, and *n* is the shape parameter. The *n* value provides an indication of the shape of the response curve. If *n* > 1, the curve is convex (it forms a shoulder-shaped response), if *n* < 1, the curve is concave (it forms a tail-shaped response), and if *n* = 1, the curve is a straight line and can be described by a linear model.

### Statistical analysis

Three replicates per treatment were used to determine how well the model fit the experimental data by calculating the Adj. R^2^ defined as follows:

4 $$ \mathrm{A}\mathrm{d}\mathrm{j}.\ {R}^2\kern0.5em  = \left[1\kern0.5em -\kern0.5em \frac{\left(m-1\right)\left(1\kern0.5em -\kern0.5em \frac{SSQregression}{SSQtotal}\right)}{m\kern0.5em -\kern0.5em j}\right] $$

where *m* is the number of observations, *j* is the number of model parameters, and SSQ is the sum of squares.

The effect of different additives on the kinetic parameters and survival of virus was assessed by using a mixed model (SAS, v9.3; SAS Inst. Inc., Cary, NC) that included the effect of additives and time as fixed effects and replicate/batch as random effects. Each vial was considered as the experimental unit. Data were analyzed for outliers and the presence of a normal distribution using the UNIVARIATE procedure of SAS that calls for calculations of sample moments, measurements of location and variability, standard deviation, test for normality, robust estimates on scale, missing values among others. The LSMEANS statement in SAS was used to calculate treatment means adjusted for model effects, while Tukey’s test was used to determine differences among treatments. For this study, significance was considered when *P* < 0.05.

## Results

### Effect of additives on the survival of PDCoV in feed at their recommended concentrations

The goodness of model fit was analyzed by comparing the Adj. R^2^ values from the log-linear and Weibull models. The Adj. R^2^ values for the log-linear model (0.48–0.57) were less than those obtained for the Weibull model (0.86–0.93), indicating that the Weibull model provide a better fit of the experimental data (Table 2). This is explained mainly because the appearance of a resistant fraction of the virus that was able to survive longer than the length of the experiment (35 days). This residual survival produced long tails in the survival curves characterized by shape parameters (n) less than 1. This nonlinear behavior resulted in D-values that overestimated virus survival (14.13–15.52 days), while the delta values obtained with the Weibull model were between 0.86 and 1.72 days. Weibull prediction values showed much faster inactivation kinetics and thus characterized better the virus survival curves.

**Table 2:** Kinetic parameters and correlation coefficients corresponding to the log-linear and Weibull models fitted to survival curves of Porcine Delta coronavirus (PDCoV) in complete feed and feed additives included at the manufacturers’ recommended concentrations

		Log-linear model		Weibull model		
Additive^1^	Log reduction (35 days)	D-value	Adj R^2^	Delta (days)	Shape parameter (n)	Adj R^2^
Control	3.0	14.73 ± 1.04	0.55	0.86 ± 0.64	0.27	0.92
UltraAcid P	3.0	15.52 ± 2.09	0.48	0.62 ± 0.56	0.23	0.89
Acid Booster	3.0	14.41 ± 0.90	0.57	1.72 ± 1.85	0.32	0.86
KEMGEST	3.0	14.73 ± 1.04	0.55	0.86 ± 0.64	0.27	0.92
Activate DA	3.0	14.73 ± 1.04	0.55	0.86 ± 0.64	0.27	0.92
Luprosil	3.0	14.13 ± 0.90	0.55	1.00 ± 0.79	0.29	0.93
Formic Acid	3.0	14.73 ± 1.04	0.55	0.86 ± 0.64	0.27	0.92
Sugar	3.0	14.73 ± 1.04	0.55	0.86 ± 0.64	0.27	0.92
Salt	3.0	14.41 ± 0.90	0.57	1.70 ± 1.85	0.32	0.89

^1^UltraAcid P, (Nutriad, Dendermonde, Belgium), Activate DA (Novus International, St. Charles, MO), Acid Booster (Agri-Nutrition, DeForest, WI), Kemgest (Kemin Agrifoods, Des Moines, IA), Luprosil (BASF, Florham Park, NJ), and formic acid (BASF, Florham Park, NJ)

In spite differences in virus inactivation kinetics, none of the additives appear to be effective in completely inactivating the virus. The total amount of virus inactivation over the sampling period of 35 days was 3 log reduction for the control sample and all the additives evaluated, indicating that none of the additives added at the manufacturers’ recommend doses were effective in reducing PDCoV survival.

### Effect of additives on the survival of PDCoV in feed at twice the recommended concentration

Doubling the concentrations of feed additives resulted in faster PDCoV inactivation kinetics (0.0004–0.28 days) for all additives, except for sucrose and formic acid (Table 3). UltraAcid P and KEMGEST provided faster initial virus inactivation kinetics than the other additives, and the delta values were estimated to be 35 s. However, most of the survival curves suggested that a large fraction of the virus remained resistant to the treatment with the appearance of tails (n values < 1) and a maximum inactivation degree achieved of 2 log after 10 days of storage. The addition of Luprosil (0.06 days), Acid Booster (0.28 days), and sodium chloride (0.09 days) resulted in the greatest virus inactivation with 2.3-3.0 log reduction after 10 days of storage at room temperature.

**Table 3:** Kinetic parameters and correlation coefficients corresponding to the Weibull model fitted to PDCoV survival curves in complete feed and feed additives that were added at twice the manufacturers recommended concentrations

		Log-linear model		Weibull model		
Additive^1^	Log reduction (10 days)	D-value^1^	Adj R^2*^	Delta^2^ (days)	Shape parameter (n)	Adj R^2*^
Control	2.0	6.05 ± 0.00	0.46	0.35^be^ ± 0.00	0.23	0.86
UltraAcid P	2.0	7.42 ± 0.00	0.22	0.0004^a^ ± 0.00	0.05	0.99
Acid Booster	2.7	4.65 ± 1.24	0.59	0.28^be^ ± 0.18	0.27	0.93
KEMGEST	2.0	7.42 ± 0.00	0.22	0.0004^a^ ± 0.00	0.05	0.99
Activate DA	2.0	6.74 ± 0.60	0.18	0.12^bd^ ± 0.20	0.13	0.72
Luprosil	2.3	4.97 ± 2.40	0.27	0.06^b^ ± 0.03	0.13	0.69
Formic Acid	2.0	8.52 ± 0.00	0.08	4.95^ac^ ± 0.00	0.02	0.50
Sugar	2.0	10.00 ± 0.00	0.13	4.94^ac^ ± 0.00	0.07	0.17
Salt	3.0	4.41 ± 0.52	0.55	0.09^bd^ ± 0.02	0.22	0.91

^1^UltraAcid P, (Nutriad, Dendermonde, Belgium), Activate DA (Novus International, St. Charles, MO), Acid Booster (Agri-Nutrition, DeForest, WI), Kemgest (Kemin Agrifoods, Des Moines, IA), Luprosil (BASF, Florham Park, NJ), and formic acid (BASF, Florham Park, NJ)

^a, b, c, d^Means of 3 replications; different superscripts differ at (*P <* 0.05)

^e^Trend comparing 2× Acid Booster vs. control (*P <* 0.1)

**Fig. 1:**
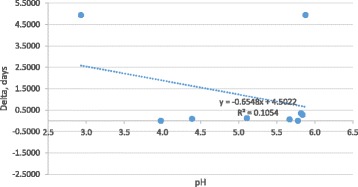
Correlation of pH and delta value on virus inactivation at double the recommended concentration of feed additives

The pH of the complete feed without addition of acidifiers was greater than pH of the same complete feed with the addition of Luprosil, Activate DA, KEMGEST, Acid Booster, and Amasil. The pH of the complete feed with addition of UltraAcid P was not different from that of the complete feed. There was no correlation between the pH values of the diet with the addition of acidifiers and the inactivation kinetics of PDCoV (delta values; Fig. 1). Interestingly, the virus appeared to survive better at pH values lower than 3 and at pH 7 to 8.

## Discussion

Organic, inorganic, or blends of acids are commonly added to swine feeds to control pathogens such as *Salmonella* spp. [13]. To our knowledge, this is the first study that has evaluated the impact of commercially available acids, sodium chloride, and sucrose on the survival of PDCoV in swine feed. When these commercial additives were added at the manufacturers’ recommended doses, none of them were effective in decreasing survival of PDCoV, we had to add all acidifiers at twice the manufacturer recommended concentrations to observe inactivation of PDCoV in complete swine feed. In contrast, PEDV is inactivated by similar acidifiers at the manufacturers’ recommended concentration; Activate DA (0.81 d) and KEMGEST (3.28 d) produced inactivation PEDV that was faster than inactivation in the control diet [14].

The current experiment focused on determining inactivation kinetics of commercial additives available to the United States feed industry, and did not focus on evaluating the specific active ingredients present in these additives that may inactivate PDCoV. However, based on the description and order of the active ingredients listed for each commercial additive, it appears that some form of phosphoric acid (pKa 6.9 × 10 ^−3^) was present in UltraAcid P and KEMGEST, which suggests that this acid may be potentially responsible for inactivation of PDCoV. Phosphoric acid has been shown to inactivate pathogens such as *Salmonella* spp. on stainless steel surfaces, but there are no data available on inactivation of viruses in animal feed [15].

Inactivation of PDCoV was greater in the presence of KEMGEST than Acid Booster, but the active ingredients in these two feed additives are similar, with the exception of fumaric acid present in KEMGEST. Furthermore, fumaric acid was also present in UltraAcid P, which was also effective in rapidly inactivating PDCoV. Therefore, it is possible that fumaric acid in KEMGEST and UltraAcid P may be the primary component that causes PDCoV inactivation. Studies have shown that fumaric acid is an effective antimicrobial that reduces survivability of *E. coli* [16] and *Salmonella* spp. [17]*.* It is believed that changes in pH affect viruses by increasing sensitivity to deoxyriobonuclease [18] and by altering the virus capsid by the loss of structural proteins [18]. The RNA of RNA-containing viruses (such as PDCoV) is sensitive to ribonuclease at all pH levels tested (pH 3–9) [19]. At pH levels of 5 and 7, RNA was hydrolyzed and there was an absence of ribonuclease. There is no clear pattern or indication of a specific acid that inactivates PDCoV and more research is needed to depict the acid or combination of acid that can completely inactivate the virus.

Comparing data from this experiment with data on inactivation of PEDV, it appears that PDCoV is more labile than PEDV to environmental temperature and storage conditions because the delta values for PDCoV were, in general, much less (<2 d) than 17 days observed for PEDV [20]. Comparison of inactivation kinetics suggest that PEDV resists inactivation during feed storage to a greater extent than does PDCoV. There are limited data comparing the survival of enteric coronaviruses in the environment, but after the initial outbreak of each virus, PEDV infected more number of herds than PDCoV, this epidemiology and geographic distribution data suggest that PEDV survives longer than PDCoV and in agreement with observations of the current experiment [21, 22].

Addition of salt, but not sugar, to the control diet caused a decrease in delta values for inactivation of PDCoV. This observation is in agreement with inactivation of PEDV in complete swine feed, where adding both salt and sugar increased inactivation of PEDV [20]. Likewise, this observation is in agreement with results from an experiment that suggest that addition of phosphate supplemented salt mix to casting for sausage manufacturing increases inactivation of several viruses affecting swine such as Food and Mouth Disease Virus, Classical Swine Fever Virus, Swine Vesicular Disease Virus, and African Swine Fever Virus [23].

## Conclusions

Using feed acidifiers could be an effective strategy to decrease the concentration of PDCoV in swine feed, but double the manufacturer’s recommended concentration was required to observe an effect. Using feed acidifiers could be an effective strategy to decrease the concentration of PDCoV in swine feed, but double the manufacturer’s recommended concentration was required to observe and effect. In spite the observed results on inactivation of PDCoV more experiments are needed to demonstrate the effectiveness of these treatments as means of preventing PDCoV transmission in feed on more applied settings. None of the treatments applied in this experiment were completely effective in inactivating PDCoV. Therefore, the strategy proposed in this research should be used in combination with other virus inactivation procedures within the processing and distribution steps for swine feed rather than a single kill step for virus inactivation.

## Abbreviations

Adj. R^2^: Adjusted coefficient of correlation
CP: Crude protein
DM: Dry matter
EE: Ether extract
PDCoV: Porcine delta coronavirus
PEDV: Porcine epidemic diarrhea virus
TCID: Tissue culture infectious dose
